# Comparison of a single-access glove port with a SILS™ port in a surgical simulator model using MISTELS

**DOI:** 10.1186/s12917-021-02958-y

**Published:** 2021-08-25

**Authors:** Ulrike Strohmeier, Gilles Dupré, Barbara Bockstahler, Alexander Tichy, Lea Liehmann

**Affiliations:** 1 Department of Small Animal Surgery, Ophthalmology, Dentistry and Physiotherapy, University of Veterinary Medicine Vienna, Veterinärplatz 1, 1210 Vienna, Austria; 2 Department of Biomedical Sciences, Platform Bioinformatics and Biostatistics, University of Veterinary Medicine Vienna, Veterinärplatz 1, 1210 Vienna, Austria

**Keywords:** Laparoscopy, Single port, SILS™ port, Glove port, Simulation, MISTELS

## Abstract

**Background:**

Recent advances in laparoscopy both in human and veterinary medicine have looked at means of being less invasive by using single-port access surgery as opposed to multiport access surgery. The glove port has gained popularity as a cost-effective alternative to commercially available single-port access devices. The primary aim of this study was to compare the glove port to the SILS™ port in a simulator model using the first two MISTELS (McGill inanimate system for training and evaluation of laparoscopic skills) tasks (peg transfer and pattern cutting).

**Methods:**

Twenty-two novices were enrolled in this experimental study. Each participant had 60 min to practise both MISTELS tasks using two-port laparoscopy. Thereafter participants performed both tasks using the glove and SILS™ port with scores being calculated based on task completion time and errors. Higher scores were indicative of better performance. Participants were assigned into two groups with the starting order of the single ports being randomly selected. A self-evaluation questionnaire with three questions was completed by each participant after testing, rating each port.

**Results:**

Significantly (*p* < 0.05) higher scores were achieved using the glove port compared to the SILS™ port when performing both tasks. The glove port was subjectively evaluated as easier to use with more manoeuvrability of the instruments than the SILS™ port.

**Implications of the study:**

The glove port’s improved manoeuvrability and ease of use make it a cost-effective alternative to the SILS™ port, for use in single-port laparoscopic veterinary surgery.

## Backround

**Fig. 1:**
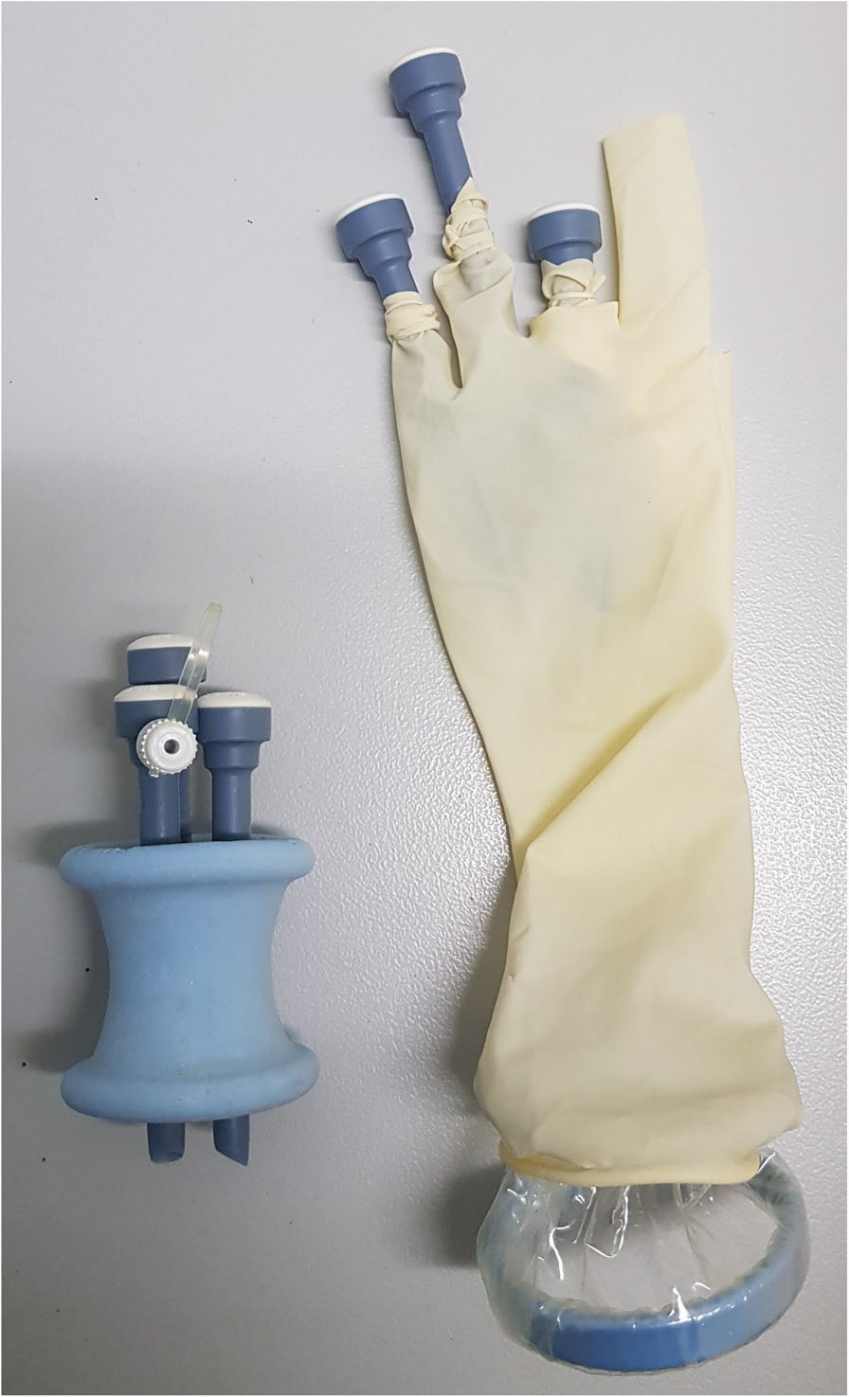
The left of the image is the SILS™ port and to the right of the image is the glove port. An ALEXIS® wound protector retractor is attached to a glove with slim trocars placed through holes made in the fingers of the glove

The SILS™ port (Medtronic Austria GmbH) is a commercially available hourglass-shaped, single-access port that is made from an elastic polymer. It contains four openings: one for insufflation and three for trocars of 5–12 mm in size as shown in Fig. 1 [1, 2]. The glove port is a laparoscopic single-incision port that is made with an ALEXIS® wound protector retractor (Applied Medical, Salzburg, Austria) to which a set of sterile gloves is attached, and slim trocars are placed through holes made in the fingers of the glove [3–8] as shown in Fig. 1.

The glove port was first described in 2009 and 2010 in various human urological [9], general surgical [5, 10], and gynaecological procedures [11]. Since its inception, various modifications have been made, such as replacing the ALEXIS® wound retractor with a flexible ring [4], or making 2.5–3 mm holes in the glove fingers and directly inserting the instruments rather than using trocars [6]. This glove port was developed for use in developing countries as a cost-effective alternative to commercially available single-port access devices [4, 9, 12, 13]. More recently, the glove port has also been described for paediatric appendectomies [13]. In veterinary surgery, the glove port has been described in elective canine laparoscopic-assisted ovariohysterectomies [8] and in canine pyometra cases [7].

One advantage of the SILS™ port and glove port is their compressibility, allowing a snug fit to the abdominal wall via a 2–3 cm mini laparotomy. This in turn reduces carbon dioxide leakage [3] and likely slightly compensates for the triangulation difficulties compared to more rigid single-access port devices [1]. This relatively simple insertion technique without prior insufflation may also reduce the risk of iatrogenic complications associated with Veress needle, direct trocar insertion, and Hasson entry techniques [14, 15].

Minimally invasive surgery (MIS) has become the standard of care in many human surgical procedures [16, 17]. Laparoscopy, however, requires specific hand - eye coordination and depth perception skills, which are quite different from the skills learnt in conventional open surgery [18–23]. In order to improve the safety of patients undergoing laparoscopic procedures, much emphasis has been placed on developing and validating simulation-based training models in the past two decades [17, 24, 25].

The fundamentals of the laparoscopic surgery (FLS) programme were first introduced in 2004 by the Society of American Gastrointestinal Endoscopic Surgeons in the form of an educational programme designed to teach and assess skills unique to laparoscopy [18, 19, 24, 26–29]. The manual skills component of the FLS programme encompasses five tasks with an objective scoring system, which is better known as MISTELS (McGill inanimate system for training and evaluation of laparoscopic skills) [18, 19, 24, 27–29]. In 2009, the American board of surgery made successfully completing an FLS programme a standard requirement for residents sitting their qualifying examination [29]. Similarly, in veterinary surgery, a veterinary assessment of laparoscopic skills (VALS) programme using MISTELS has been developed to assess and effectively evaluate skills [22, 25, 30].

More recent advances in laparoscopy both in human and veterinary medicine have looked at means of being even less invasive by using single-port access surgery (SPAS) as opposed to multiport access surgery [31–34]. Reducing the number of incisions and trocar insertions into the abdomen not only reduces patient morbidity but also improves cosmesis [1, 3, 4, 12, 33]. SPAS is, however, more technically challenging due to increased instrument collisions both inside and outside the abdomen due to their common entry point, known as “sword fighting” [35] and the difficulty in triangulation [3, 31, 32, 36]. To this end, multiple specifically manufactured single-access devices have been developed for commercial use, such as the EndoCone™ and S-Ports (Karl Storz Endoscopy), the GelPoint Access System (Applied Medical Inc.), the SILS™ port (Medtronic), Triport (Olympus), and AirSeal (SurgiQuest) to name but a few [2, 3]. Specially designed articulating and rotating instruments have also been produced to help overcome the triangulation and ergonomic difficulties associated with single-port surgery [2, 3, 37].

In a recent experimental study at this institution, the manoeuvrability of scopes and instruments within three different single-port access systems (SPAS) was compared [38]. The EndoCone™, SILS™ port, and glove port were alternately placed in an acrylic box with a 10 mm endoscope and a 5 mm laparoscopic instrument [38]. A motion analysis system with 20 cameras was used to track the motion of different combinations of either the endoscope alone or the endoscope and the instrument inside the three SPAS [38]. The area of manoeuvrability was defined as the conic section seen by the endoscope [38]. The authors found the area of manoeuvrability to be significantly higher when using the glove port (2.161 cm^2^) compared to the SILS™ port (867 cm^2^) or EndoCone™ (87 cm^2^) [38].

The primary aim of this study was to compare the glove port to the SILS™ port using the task scores achieved by novices in a simulator model when performing the first two MISTELS tasks (peg transfer and pattern cutting). Our hypothesis was that due to the improved manoeuvrability afforded by the glove port [38], the tasks scores achieved for both exercises would be higher using this port when compared to the SILS™ port.

## Methods

### Participants

Ethical approval for the study was requested from the institutional review board of the Medical University of Vienna, prior to commencement of the study. The review board stated that ethical approval was not required for this study. Written consent to participate in the study and permission to publish the results in accordance with data protection laws was obtained from each participant. Twenty-two participants were voluntarily enrolled in the project. Included participants were either veterinary surgeons working at the University of Veterinary Medicine Vienna or veterinary students in their final year of study. To be included participants had to be novices which was defined has having no prior experience in performing laparoscopic surgical procedures.

### Instrumentation and simulator

**Fig. 2:**
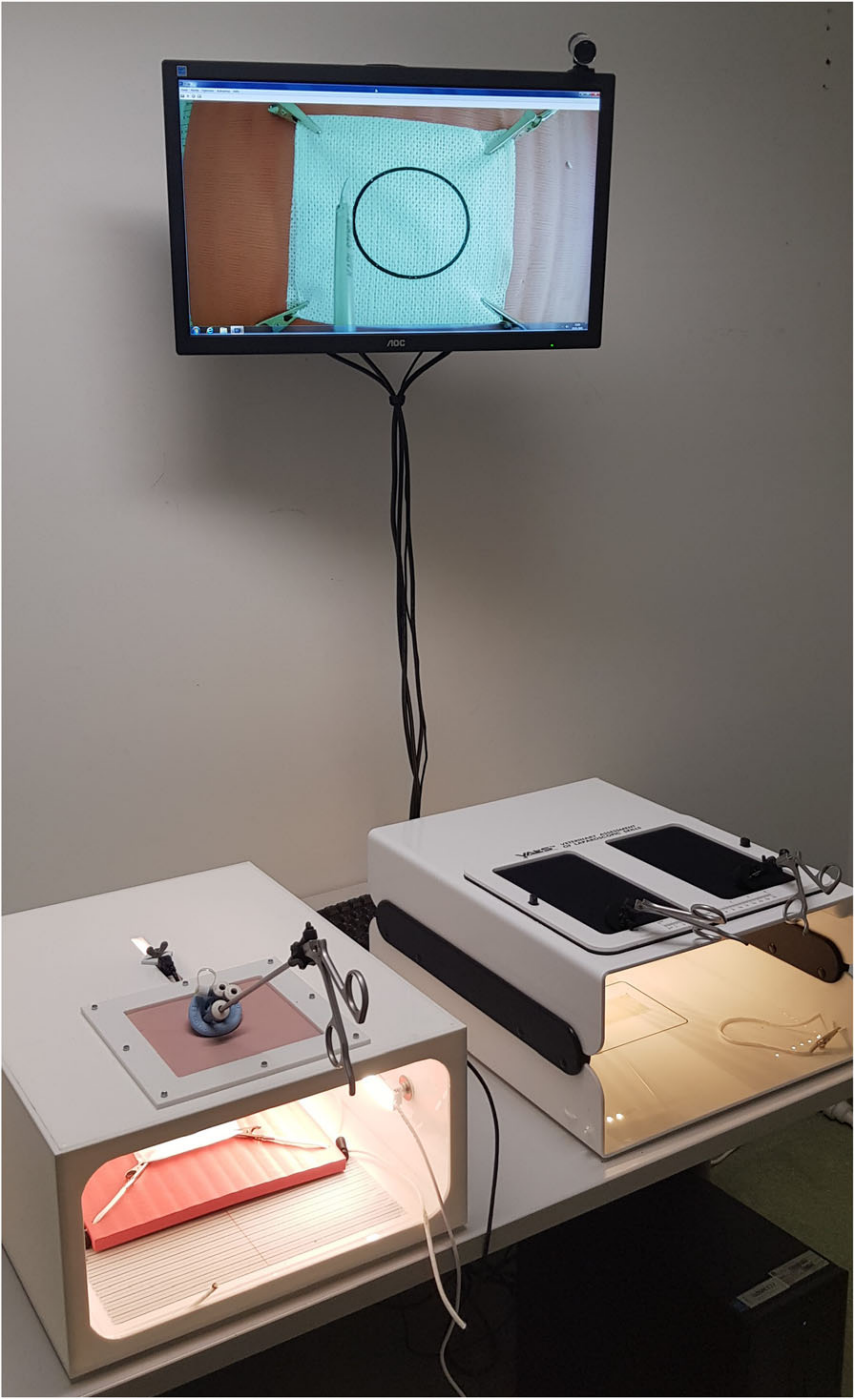
Official VALS box trainer on the right of the image and single access port box trainer with attached SILS™ port on the left of the image

**Fig. 3:**
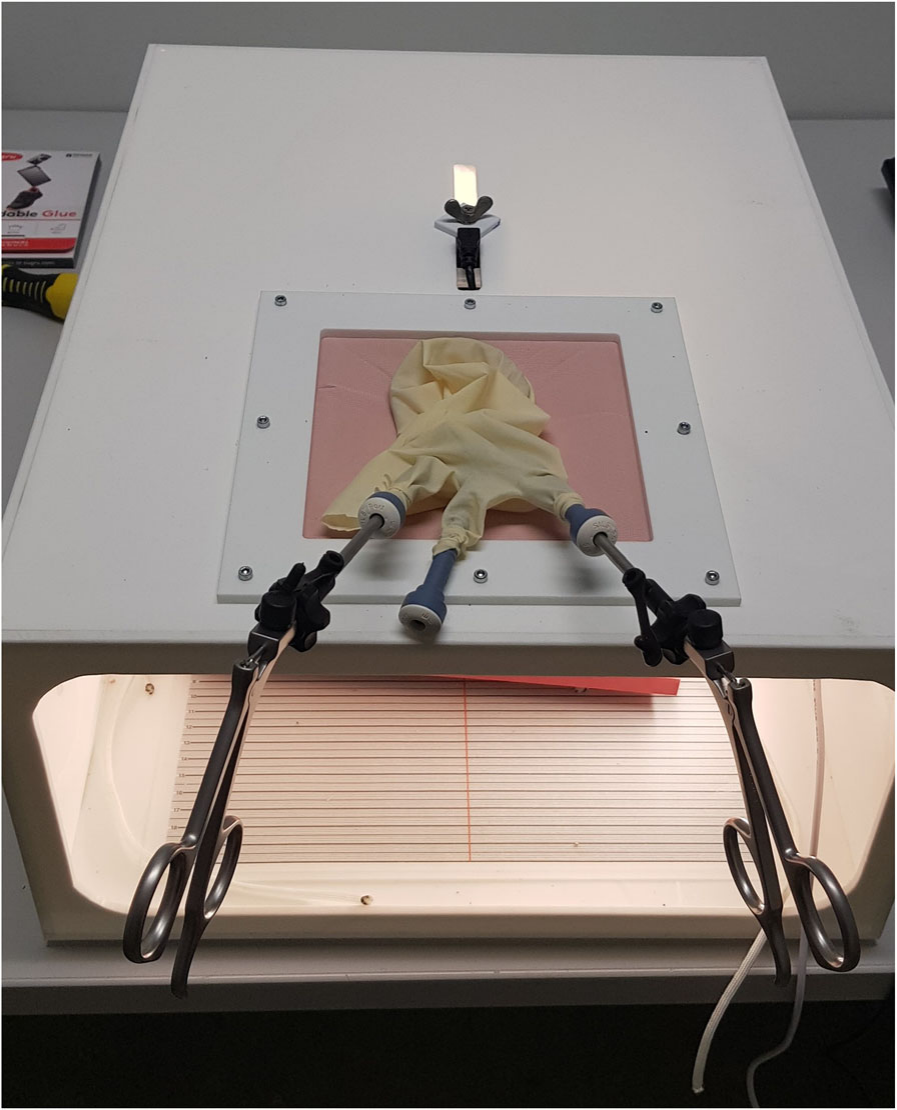
Single access port box trainer with attached glove port

This experimental study was performed in a dedicated room with two laparoscopic box trainers, each set up with a high-definition camera connected to a single computer monitor Fig. 2. The box trainer with two access ports is the official VALS box trainer [39, 40] with a skin frame constructed from durable Neoprene and two pre-incised holes for trocars. The other simulator box was built specifically for this experiment. The built box trainer was fitted with a square laparo-abdominal pad (limbs and things, Bristol, UK) into which a 2.5 cm diameter centred hole was cut to fit the single-access port devices. The centred hole could accommodate either the SILS™ port (Medtronic, Austria GmbH) or the glove port see Fig. 3.

The modified glove port was based on the one described by Khiangte et al. [4] and its assembly closely followed the descriptions by Becher-Deichsel et al. [7] and Bydzovsky et al. [8]. Briefly, an XS ALEXIS™ (Applied Medical, Salzburg, Austria) wound retractor was attached to a size 6 ½ surgical glove (Vasco OP sensitive; B. Braun Melsungen AG, Melsungen, Germany). The inner ring of the wound retractor was placed through the centred hole and the outer ring was then rolled inwards until the retractor remained taut. Three 5 mm holes were made in the index, middle, and little fingers of the glove and three 5 mm cannulas (SILS™ port, Medtronic, Austria GmbH) were then secured into these holes using an elastic tie cut from another size 6 ½ glove. The wrist part of the glove was then secured over the outer ring of the wound retractor [7].

Each single-access port was fitted with three 5 mm reusable cannulas (Medtronic, Austria GmbH) [7, 8, 31, 36]. Two straight 5 mm laparoscopic grasping forceps (Karl Storz Endoskop, Austria GmbH) were used for the peg transfer task [17, 31, 32, 41]. A pair of straight 5 mm grasping forceps and a pair of straight 5 mm laparoscopic scissors (Karl Storz Endoskop, Austria GmbH) were used for the pattern cutting task [22, 25, 30, 32]. The third cannula was left empty during both tasks.

### MISTELS tasks

Each participant had an initial training session during which a video was shown on youtube.com: the FLS expanded video tutorial Series; Task 1-Peg Transfer [42] and Task 2- Pattern Cut [43]. A proctor (US) answered any further questions and was responsible for all the testing and calculation of scores for each participant.

All participants had 60 min to practise the two MISTELS tasks using the two-port VALS laparoscopic box trainer as seen in Fig. 2. Criteria for inclusion in the study included completion of both tasks within a 600 s cut-off time using the conventional two-port model. No prior practice using the single-access ports (glove port and SILS™ port) was permitted before testing began. The testing was done in a separate box trainer with a single-access port as seen in Figs. 2 and 3. The order in which the exercises were performed using the single ports (SILS™ or glove port) was randomised, i.e., 11 participants (group 1) started with the glove port followed by the SILS™ port, and the other 11 participants (group 2) started with the SILS™ port followed by the glove port.

**Fig. 4:**
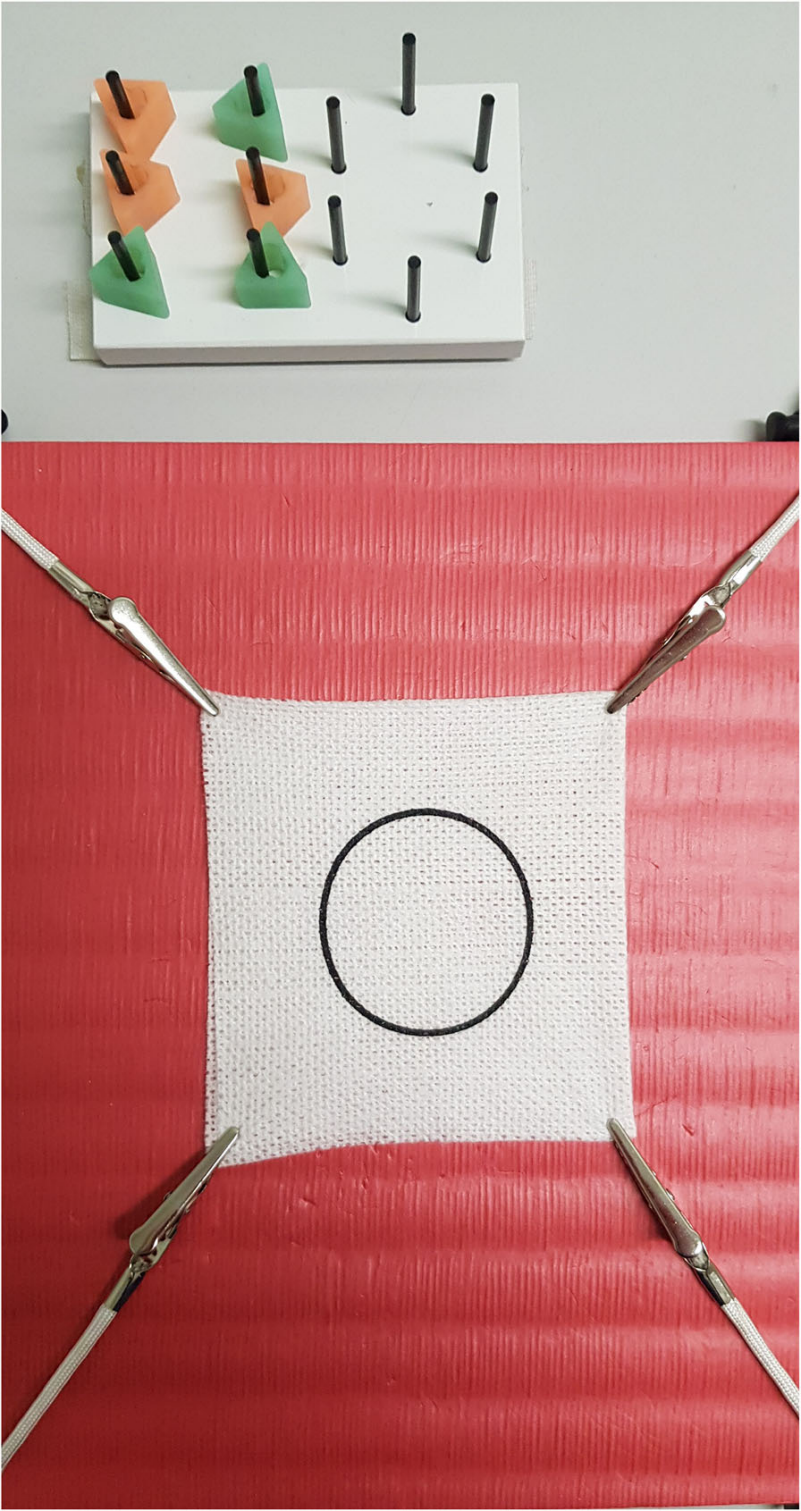
Top of the image is the Peg Board with 6 colour triangles. Bottom of the image is the gauze swab attached to a Styrofoam board by alligator clips

The peg board consisted of 12 pegs and six coloured moveable hollow triangles. On the left hand-side of the board, the six pegs were aligned in a rectangle, and on the right the pegs were aligned in a circle (Fig. 4). The exercise was started with all six triangles on the left-hand side of the peg board, in the rectangle. The triangles were first picked up with a grasper using the non-dominant hand, moved mid-air to the dominant hand, and then placed on the pegs on the right-hand side, on the circular part of the board. The exercise was then reversed, with the triangles being moved from the right circular part to the left rectangular part of the board. During the reverse of the exercise the dominant hand grasper was used to pick up a triangle on the right, transferred mid-air to the non-dominant hand grasper, and placed on the left-hand side of the board. The order in which the triangles were picked up was irrelevant.

For the pattern-cutting task, a 10 × 15 cm surgical gauze swab with a pre-printed 5 cm diameter circle drawn in the centre was used. This swab was fixed to a Styrofoam board with alligator clips (Fig. 4). The circle was cut out using a pair of laparoscopic scissors in the dominant hand whilst holding the swab under tension with laparoscopic graspers in the non-dominant hand.

### Scoring

The scoring system used was adapted from Chen et al. 2019 [44] and Brown-Clerk et al. 2011 [31] using task completion time and number of errors.

### Scoring for the peg transfer task

The raw score was calculated as follows:

$$ Raw\  score=\left\{\left(600\  seconds- time\ to\ completion\ in\ seconds\right)-\left(50\ x\  number\ of\ dropped\ pegs\  on\  the\ first\ transfer\right)+\left(25\ x\  number\ of\ dropped\ pegs\  on\  the\ second\ transfer\right)\right\}. $$

A score of zero was given if the task took longer than 600 s to complete. This raw score was then divided by 237 and multiplied by 100 to obtain a normalised score. The figure of 237 is a predetermined standard value derived from the maximum score achieved by a chief surgical resident in the FLS programme [19, 28, 41].

### Scoring for the pattern cutting task

The raw score for the pattern cutting task was calculated as follows:

$$ Raw\  score=\left\{\left(600\  seconds- time\ to\ completion\ in\ seconds\right)-\left(\% deviation\ from\ the\  pre- marked\ circle\ in\ {mm}^2\right)\right\} $$

The percentage deviation was calculated by laying out any excess swab material resulting from cut deviations outside or inside the perfect circle on graph paper. The area to the nearest rectangle was then calculated. This rectangle area was then divided by the circle area to determine the percentage deviation.

A score of zero was given if the task took longer than 600 s to complete. This raw score was then divided by 280 and multiplied by 100 to obtain a normalised score. The figure of 280 is a predetermined standard value derived from the maximum score achieved by a chief surgical resident in the FLS programme [19, 28, 41].

### Self-evaluation

A subjective self-evaluation questionnaire was completed by each participant following completion of the tasks. In this questionnaire, each port’s manoeuvrability, task completion difficulty, and ease of use was rated according to a Likert scale from 1 (exceedingly difficult) to 3 (extremely easy) (Table 1).

**Table 1:** Self-evaluation questionnaire

**Question 1:** How difficult was it to complete the peg transfer task using each port on a scale of 1–3? Rated: 1 (exceedingly difficult), 2 (neither difficult nor easy), 3 (extremely easy)			
	1 (exceedingly difficult)	2 (neither difficult nor easy	3 (extremely easy)
SILS™ Port			
Glove Port			
**Question 2:** How difficult was it to complete the pattern cutting task using each port on a scale of 1–3? Rated: 1 (exceedingly difficult), 2 (neither difficult nor easy), 3 (extremely easy)			
	1 (exceedingly difficult)	2 (neither difficult nor easy	3 (extremely easy)
SILS™ Port			
Glove Port			
**Question 3:** How difficult was it to manoeuvre the instruments inside each port on a scale of 1–3? Rated: 1 (exceedingly difficult), 2 (neither difficult nor easy), 3 (extremely easy)			
	1 (exceedingly difficult)	2 (neither difficult nor easy	3 (extremely easy)
SILS™ Port			
Glove Port			

### Statistical analysis

Statistical analysis was performed using the IBM SPSS v24. Descriptive data are reported as mean and standard deviation (SD). The assumption of a normal distribution was tested using the Kolmogorov -Smirnov -test. A general linear model was used to analyse the differences in scores between the two ports where the ports were added as a within-subject factor. To control for the different sequences in the crossover design, the sequence was added as a between-subject factor. Post hoc tests were applied using Bonferroni’s alpha correction procedure. The three questions of the self-evaluation questionnaire were analysed using the Wilcoxon signed-rank test to compare the two ports. A *p* value of < 0.05 indicated statistical significance.

## Results

Twenty veterinarians and two final year veterinary students participated in the study. Fifteen participants were female and seven participants male. Eleven participants (group 1) started with the glove port, followed by the SILS™ port, and the other 11 participants (group 2) started with the SILS™ port followed by the glove port.

### Peg transfer results

**Fig. 5:**
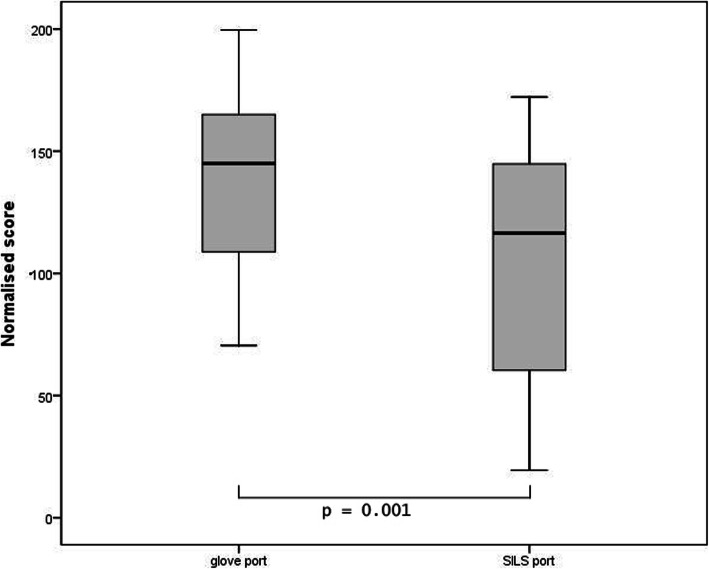
Box plot graph of the peg transfer task comparing the normalised scores achieved by the 22 participants using both the glove port and the SILS™ port. The Y-axis represents the normalised scores. The boxes represent values between the first quartile (Q1) and the third quartile (Q3). Whiskers ranging from Q1 to the minimum and from Q3 to the maximum. The bold line shows the median. The first box plot on the X-axis represents the glove port and the second box plot represents the SILS™ port. The p-value indicates the significance of the difference between the two ports using the general linear model

The general linear model showed significant score differences between the two ports (*p* = 0.01) and no significant interaction with the starting order could be found (*p* = 0.745). Participants achieved higher scores using the glove port (mean = 138.1; SD = 38.8) compared to using the SILS™ port (mean = 106.7; SD = 46.8). The mean results of both groups are summarized in Table 2. Table 3 shows the 95% confidence intervals of the total scores. A graphic representation of the scores achieved by the participants is shown in Fig. 5.

**Table 2:** Presentation of the normalised scores achieved when performing the peg transfer and pattern cutting tasks using the two ports

Ports and groups		Peg transfer task		Pattern cutting task	
Ports	Groups	Mean score	Standard deviation	Mean score	Standard deviation
glove port	group 1	119.6	35.1	99.3	32.2
	group 2	156.4	34.5	136.7	25.8
	total	138.1	38.8	118.1	34.3
SILS™ port	group 1	91.9	45.3	94.4	54.1
	group 2	121.4	45.5	104.3	39.9
	total	106.7	46.8	99.4	46.7

**Table 3:** Mean scores and confidence intervals for both tasks

**Peg transfer**	**Mean**	**Standard error**	**95% confidence interval lower limit**	**95% confidence interval upper limit**
glove	138.1	7.4	122.6	153.5
SILS™	106.7	9.7	86.5	126.9
**Pattern cutting**	**Mean**	**Standard error**	**95% confidence interval lower limit**	**95% confidence interval upper limit**
glove	118.1	6.2	105.1	131.0
SILS™	99.4	10.1	78.2	120.6

### Pattern cutting results

**Fig. 6:**
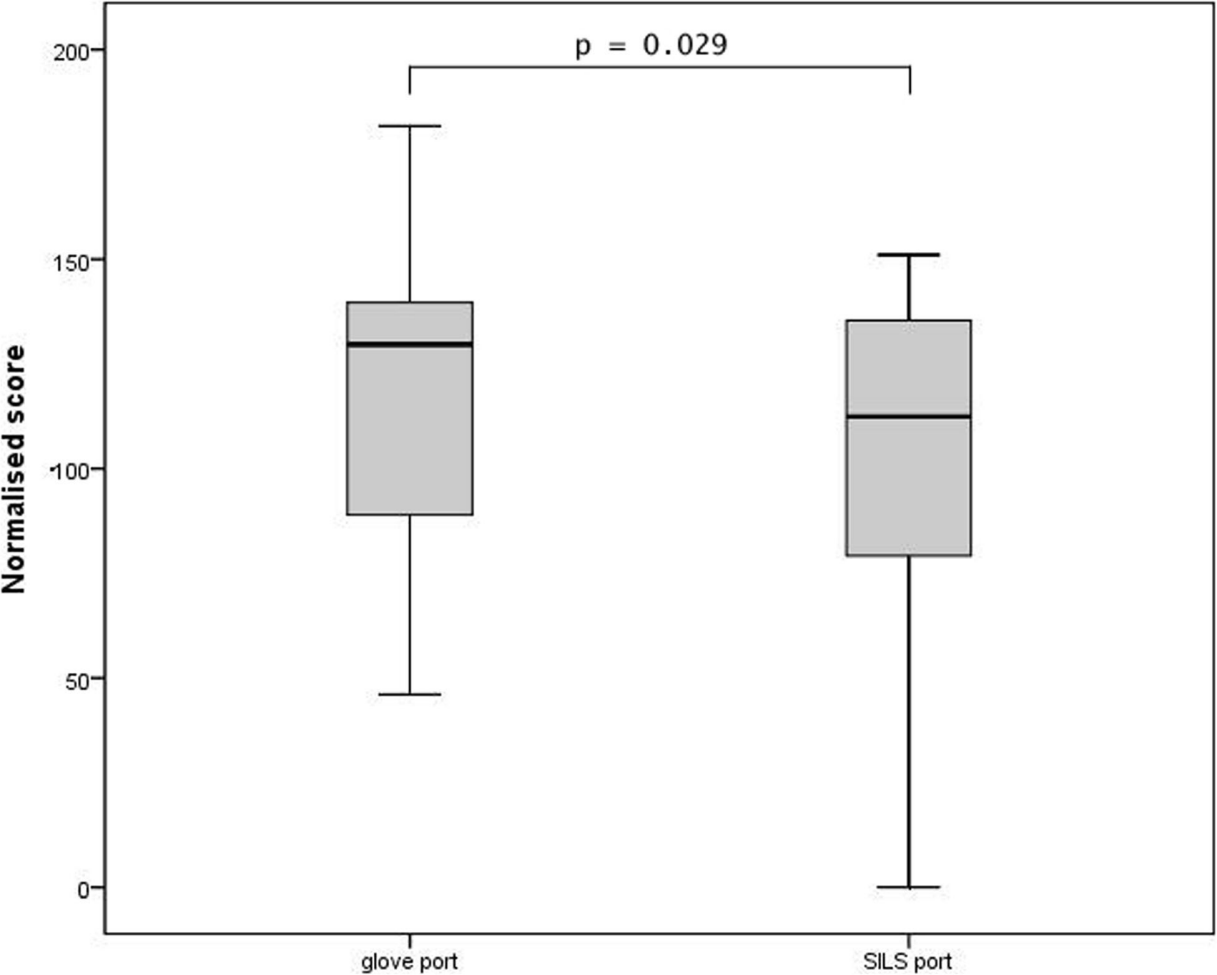
Box plot graph of the pattern cutting task comparing the normalised scores achieved by the 22 participants using both the glove port and the SILS™ port. The Y-axis represents the normalised scores. The boxes represent values between the first quartile (Q1) and the third quartile (Q3). Whiskers ranging from Q1 to the minimum and from Q3 to the maximum. The bold line shows the median. The first box plot on the X-axis represents the glove port and the second box plot represents the SILS™ port. The *p*-value indicates the significance of the difference between the two ports using the general linear model

The general linear model showed significant score differences between the two ports (*p* = 0.029) and no significant interaction with the starting order could be found (*p* = 0.098). Participants achieved higher scores using the glove port (mean = 118.1; SD = 34.3) compared to the SILS™ port (mean = 99.4; SD = 46.7). The mean results of both groups are summarized in Table 2. Table 3 shows the 95% confidence intervals of the total scores. A graphic representation of the scores achieved by the participants is shown in Fig. 6.

### Self-evaluation questions

According to the Wilcoxon signed-rank test, for all three questions the SILS™ port was evaluated as significantly more difficult to use than the glove port (question 1: *p* = 0.005; question 2: *p* = 0.033; question 3: *p* = 0.035). The glove port was rated as neither difficult nor easy to use by most respondents whereas the SILS™ port was most frequently rated as exceedingly difficult to use by most respondents. The responses of the participants to the three questions is depicted in Table 4.

**Table 4:** Self-evaluation results

Questions			Glove port			Total
			Very difficult	Neither difficult nor easy	Very easy	
**1**	**SILS port**	**exceedingly difficult**	2	10	1	13
		**neither difficult nor easy**	2	4	2	8
		**very easy**	0	0	1	1
	**Total**		4	14	4	22
**2**	**SILS port**	**exceedingly difficult**	3	15	0	18
		**neither difficult nor easy**	2	0	0	2
		**very easy**	1	1	0	2
	**Total**		6	16	0	22
**3**	**SILS port**	**exceedingly difficult**	3	10	1	14
		**neither difficult nor easy**	2	3	2	7
		**very easy**	1	0	0	1
	**Total**		6	13	3	22

## Discussion

In this experimental study, participants were able to complete both MISTELS tasks in a shorter time and with fewer errors, i.e., achieve higher scores when using the glove port compared to the SILS™ port hence we accept our hypothesis. The lower scores achieved when using the SILS™ port could be explained by the proximity of the instruments due to the reduced working space, as was shown by the reduced area of manoeuvrability in the study by Haider et al. [38]. Reduced working space limits the range of motion and the ability to triangulate, and both circumstances likely cause more instrument collisions [1, 45] and sword fighting [3, 8, 35]. These increased instrument collisions would likely lead to an increase in errors and prolong the time required to complete the peg transfer and pattern cutting tasks.

Additionally, in the subjective evaluation of both tasks, participants rated the glove port as easier to use with more manoeuvrability of the instruments than the SILS™ port. This is based on the respondent’s answers to the three questions. For question one, which was related to the difficulty of completing the peg transfer task, 14/22 respondents evaluated the glove port as neither difficult nor easy whereas 13/22 respondents evaluated the SILS™ port as exceedingly difficult to use. For question two, which was related to the difficulty of completing the pattern cutting task, 16/22 respondents evaluated the glove port as neither difficult nor easy, whereas 18/22 evaluated the SILS™ port as exceedingly difficult to use. For question three, which was related to the overall manoeuvrability of the instruments within each port, 13/22 respondents evaluated the glove port as neither difficult nor easy, with 14/22 respondents evaluating the SILS™ port as exceedingly difficult to use. A similar questionnaire was used in the study by Brown-Clerk et al. [31]. In that study conventional two-port laparoscopy was rated as the easiest to use by participants, followed by the GelPoint system in second place [31]. The authors of that study also concluded that the GelPoint (single port) system appears to be the easiest system for novices to use and that it performed similarly to two-port laparoscopy [31]. Some similarities exist between the GelPoint and the glove port systems in that both constructs consist of an ALEXIS® wound retractor through which instruments are introduced. This may in part have contributed to the improved scores seen in our study.

Interestingly, no significant interaction between the starting order could be found. Participants consistently achieved higher scores using the glove port when compared to the SILS™ port, irrespective of the starting order in which they were being used.

We allowed participants to practise both tasks using two-port laparoscopy whereas no prior practice was allowed using either of the two single-access ports. We reasoned that due to the inclusion of novices and the known unique challenges associated with laparoscopy, such as the use of long instruments which amplify tremors, the limited range of motion due to placing instruments through trocars and ports, and the lack of optical depth perception [18, 22, 30], task completion would be made more difficult. Part of our inclusion criteria was that participants needed to be able to perform each task within a ten-minute cut-off time using conventional two-port laparoscopy. Single-incision laparoscopic surgery has been shown to be more technically challenging than conventional laparoscopy in a simulator model [32, 36]. In another study by Santos et al. [34], the learning curve for single-incision laparoscopy (using the peg transfer and pattern-cutting tasks) was longer than two-port laparoscopy when novices were tested. Another factor in this decision was that the primary objective of this study was to compare the task scores between the glove port and SILS™ port.

The scoring system using task completion time and number of errors for the peg transfer task was adapted from Chen et al. [44] and Brown-Clerk et al. [31]. The scoring system used for the pattern cutting task was the same as that used by Kilkenny et al. [41]. The scoring systems are adaptions from the original FLS scoring system used for human laparoscopic surgeons [19, 27, 28]. The official FLS programme and previously published veterinary studies use a 300 s cut-off time for testing the MISTELS tasks [19, 22, 25, 28, 41, 44]. The decision to allocate a ten-minute cut-off time rather than a five-minute cut-off in this study was threefold. Firstly, all participants were inexperienced in laparoscopic surgery. Secondly, single-port laparoscopy is more challenging with a steeper learning curve than standard two-port laparoscopic surgery [3]. Thirdly, we assumed that there would be significant loss of data or exclusion of participants had a 300 s cut-off time been used. The publication by Brown-Clerk et al. [31] also used a 600 s cut-off time and this study also included novices.

A limitation of this study was using a mounted camera in the box trainer rather than having an operator-driven camera as was used in the experimental model by Santos et al. [34]. Having an additional instrument would likely have led to even more instrument collisions and further reduced the available working space in the single-port access devices. This would have mimicked the intra-operative situation more closely, but it would have required an additional experienced proctor to operate the camera for each participant. We tried to negate the impact of this limitation somewhat by adding a third cannula to both single-port access devices. We recognise that an empty cannula does not wholly simulate the effect of a cannula housing an instrument, but we reasoned that the restriction in movement by an empty cannula would still be more than if the port hole had been left empty.

Straight laparoscopic graspers and shears rather than articulated instruments were used to perform both MISTELS tasks on the single-port platforms. Articulated instruments have been developed to ease the use of single-access port surgery by providing intracorporeal triangulation, decreasing instrument crowding and allowing a more ergonomic positioning of the hands [3]. In the publication by Santos et al. [34], neither the static articulating instruments nor the dynamic articulating instruments were associated with improved performance or a shorter learning curve when compared with straight instruments, when used to perform the first two MISTLES tasks in a simulator model. Straight laparoscopic instruments have been used successfully with the SILS™ port in animals undergoing laparoscopic ovariectomy [1, 46], laparoscopic-assisted gastropexy [46], and laparoscopic-assisted intestinal surgery [14]. Straight laparoscopic instruments have also been used in canines with a modified glove port undergoing an elective laparoscopic-assisted ovariohysterectomy [8] and a laparoscopic-assisted ovariohysterectomy for pyometra [7].

The findings of this study show that novices can achieve significantly higher task scores when performing the first two MISTELS tasks using the glove port compared to the SILS™ port. This cohort of participants also subjectively rated the glove port as easier to use with more manoeuvrability of the instruments in comparison to the SILS™ port.

### Implications of the study

The glove port’s improved manoeuvrability and ease of use make it a cost-effective alternative to the SILS™ port, for use in single-port laparoscopic veterinary surgery.

## Data Availability

All the relevant data generated or analysed during this study are included in the published article and further datasets are available upon request from the author, US.

## Abbreviations

MISTELS: McGill inanimate system for training and evaluation of laparoscopic skills
MIS: Minimally invasive surgery
FLS: The fundamentals of the laparoscopic surgery
VALS: Veterinary assessment of laparoscopic skills
SPAS: Single-port access surgery
US: Ulrike Strohmeier (proctor)
SD: Standard deviation

## References

[1] Manassero M, Leperlier D, Vallefuoco R, Viateau V (2012). Laparoscopic ovariectomy in dogs using a single-port multiple-access device. Vet Rec.

[2] Romanelli JR, Earle DB (2009). Single-port laparoscopic surgery: an overview. Surg Endosc.

[3] Runge Jeffrey. Single-incision laparoscopic Surgery Devices. In: Fransson, Boel. Mayhew Philipp, editor. Small Animal Laparoscopy and Thoracoscopy. Wiley Blackwell; 2015:65–71.

[4] Khiangte E, Newme I, Phukan P, Medhi S (2011). Improvised transumbilical glove port: a cost effective method for single port laparoscopic surgery. Indian J Surg.

[5] Hayashi M, Asakuma M, Komeda K, Miyamoto Y, Hirokawa F, Tanigawa N (2010). Effectiveness of a surgical glove port for single port surgery. World J Surg.

[6] Ko YS, Yoon SY, Han HJ, Yim TW, Song TJ (2015). A new glove port for single incision procedure. Ann Surg Treat Res.

[7] Becher-Deichsel A, Aurich JE, Schrammel N, Dupré G (2016). A surgical glove port technique for laparoscopic-assisted ovariohysterectomy for pyometra in the bitch. Theriogenology..

[8] Bydzovsky ND, Bockstahler B, Dupré G (2019). Single-port laparoscopic-assisted ovariohysterectomy with a modified glove-port technique in dogs. Vet Surg.

[9] Jeon HG, Jeong W, Oh CK, Lorenzo EIS, Ham WS, Rha KH (2010). Initial experience with 50 laparoendoscopic single site surgeries using a homemade, single port device at a single center. J Urol.

[10] Tai HC, da Lin C, Wu CC, Tsai YC, Yang SSD (2010). Homemade transumbilical port: an alternative access for laparoendoscopic single-site surgery (LESS). Surg Endosc.

[11] Yong WJ, Sang WK, Young TK (2009). Recent advances of robotic surgery and single port laparoscopy in gynecologic oncology. J Gynecol Oncol.

[12] Cepedal LF, Calvo MP, Ortega HM, Lasarte AS, González CP, Val JFF (2016). Glove port, how do we do it? A low-cost alternative to the single-port approach. Surg Endosc.

[13] Martynov I, Lacher M (2018). Homemade glove port for single-incision pediatric endosurgery (SIPES) appendectomy—how we do it. Eur J Pediatr Surg Rep.

[14] Case JB, Ellison G (2013). Single incision laparoscopic-assisted intestinal surgery (SILAIS) in 7 dogs and 1 cat. Vet Surg.

[15] Dupré G. Laparoscopic access techniques. In: Fransson, Boel A. Mayhew, Philipp D, editor. Small animal laparoscopy and thoracoscopy. Wiley Blackwell; 2015. p. 86.

[16] Sroka G, Feldman LS, Vassiliou MC, Kaneva PA, Fayez R, Fried GM (2010). Fundamentals of laparoscopic surgery simulator training to proficiency improves laparoscopic performance in the operating room-a randomized controlled trial. Am J Surg.

[17] Chellali A, Zhang L, Sankaranarayanan G, Arikatla VS, Ahn W, Derevianko A (2014). Validation of the VBLaST peg transfer task: a first step toward an alternate training standard. Surg Endosc.

[18] Derossis AM, Fried GM, Abrahamowicz M, Sigman HH, Barkun JS, Meakins JL (1998). Development of a model for training and evaluation of laparoscopic skills. Am J Surg.

[19] Vassiliou MC, Ghitulescu GA, Feldman LS, Stanbridge D, Leffondré K, Sigman HH (2006). The MISTELS program to measure technical skill in laparoscopic surgery: evidence for reliability. Surg Endosc Other Interv Tech.

[20] Usón-Gargallo J, Tapia-Araya AE, Díaz-Güemes Martin-Portugués I, Sánchez-Margallo FM (2014). Development and evaluation of a canine laparoscopic simulator for veterinary clinical training. J Vet Med Educ.

[21] Fransson, Boel A. Towle Millard, Heather A. Ragle CA. Surgeons’ skills training. In: Fransson, Boel A. Mayhew PD, editor. small animal laparoscopy and thoracoscopy. Wiley Blackwell; 2015. p. 3–11.

[22] Fransson BA, Ragle CA, Bryan ME (2012). Effects of two training curricula on basic laparoscopic skills and surgical performance among veterinarians. J Am Vet Med Assoc.

[23] Fransson BA, Chen CY, Noyes JA, Ragle CA (2016). Instrument motion metrics for laparoscopic skills assessment in virtual reality and augmented reality. Vet Surg.

[24] Fried GM, Feldman LS, Vassiliou MC, Fraser SA, Stanbridge D, Ghitulescu G (2004). Proving the value of simulation in laparoscopic surgery. Ann Surg.

[25] Fransson BA, Ragle CA (2010). Assessment of laparoscopic skills before and after simulation training with a canine abdominal model. J Am Vet Med Assoc.

[26] Peters JH, Fried GM, Swanstrom LL, Soper NJ, Sillin LF, Schirmer B (2004). Development and validation of a comprehensive program of education and assessment of the basic fundamentals of laparoscopic surgery. Surgery..

[27] Scott DJ, Ritter EM, Tesfay ST, Pimentel EA, Nagji A, Fried GM (2008). Certification pass rate of 100% for fundamentals of laparoscopic surgery skills after proficiency-based training. Surg Endosc Other Interv Tech.

[28] Fraser SA, Klassen DR, Feldman LS, Ghitulescu GA, Stanbridge D, Fried GM (2003). Evaluating laparoscopic skills, setting the pass/fail score for the MISTELS system. Surg Endosc Other Interv Tech.

[29] Rooney DM, Santos BF, Hungness ES (2012). Fundamentals of laparoscopic surgery (FLS) manual skills assessment: surgeon vs nonsurgeon raters. J Surg Educ.

[30] Fransson BA, Ragle CA, Bryan ME (2010). A laparoscopic surgical skills assessment tool for veterinarians. J Vet Med Educ.

[31] Brown-Clerk B, de Laveaga AE, Lagrange CA, Wirth LM, Lowndes BR, Hallbeck MS (2011). Laparoendoscopic single-site (LESS) surgery versus conventional laparoscopic surgery: comparison of surgical port performance in a surgical simulator with novices. Surg Endosc.

[32] Marcus HJ, Seneci CA, Hughes-Hallett A, Cundy TP, Nandi D, Yang GZ (2016). Comparative performance in single-port versus multiport minimally invasive surgery, and small versus large operative working spaces. Surg Innov.

[33] Dupré G, Fiorbianco V, Skalicky M, Gültiken N, Ay SS, Findik M (2009). Laparoscopic ovariectomy in dogs: comparison between single portal and two-portal access. Vet Surg.

[34] Santos BF, Reif TJ, Soper NJ, Hungness ES (2011). Effect of training and instrument type on performance in single-incision laparoscopy: results of a randomized comparison using a surgical simulator. Surg Endosc.

[35] Shussman N, Schlager A, Elazary R, Khalaileh A, Keidar A, Talamini M (2011). Single-incision laparoscopic cholecystectomy: lessons learned for success. Surg Endosc.

[36] Santos BF, Enter D, Soper NJ, Hungness ES (2011). Single-incision laparoscopic surgery (SILS™) versus standard laparoscopic surgery: a comparison of performance using a surgical simulator. Surg Endosc.

[37] Runge JJ, Mayhew PD (2013). Evaluation of single port access gastropexy and ovariectomy using articulating instruments and angled telescopes in dogs. Vet Surg.

[38] Haider G, Schulz U, Katic N, Peham C, Dupré G. Maneuverability of the Scope and Instruments within Three Different Single-Incision Laparoscopic Ports: AnExperimental Pilot Study. Animals : an Open Access Journal From MDPI. 2021;11(5). 10.3390/ani11051242. 10.3390/ani11051242 PMC8145893 33925867

[39] VALS laparoscopic skills trainer with USB camera. https://limbsandthings.com//us/products/50308/50308-vals-laparoscopic-skills-trainer-with-usb-camera/. Accessed Jan 2020.

[40] VALS equipment requirements. https://valsprogramm.org/vals-equipment-requirement/. Accessed Jan 2020.

[41] Kilkenny JJ, Singh A, Kerr CL, Khosa DK, Fransson BA (2017). Factors associated with simulator-assessed laparoscopic surgical skills of veterinary students. J Am Vet Med Assoc.

[42] st. John Surgical Residency. FLS expanded video tutorial series: task 1 - peg transfer. https://youtu.be/gAQPXHWgdXQ. Accessed Jan 2020.

[43] st. John Surgical Residency. FLS expanded video tutorial series: task 2 - pattern cutting. https://youtu.be/mUBZoSO3KA8. Accessed Jan 2020.

[44] Chen CY, Elarbi M, Ragle CA, Fransson BA (2019). Development and evaluation of a high-fidelity canine laparoscopic ovariectomy model for surgical simulation training and testing. J Am Vet Med Assoc.

[45] Tapia-Araya AE, Martin-Portugués IDG, Bermejo LF, Sánchez-Margallo FM (2015). Laparoscopic ovariectomy in dogs: comparison between laparoendoscopic single-site and three-portal access. J Vet Sci.

[46] Gonzalez-Gasch E, Monnet E (2015). Comparison of single port access versus multiple port access systems in elective laparoscopy: 98 dogs (2005-2014). Vet Surg.

